# Liposome Formulation of Fullerene-Based Molecular Diagnostic and Therapeutic Agents

**DOI:** 10.3390/pharmaceutics5040525

**Published:** 2013-10-18

**Authors:** Zhiguo Zhou

**Affiliations:** Luna Innovations Inc., 521 Bridget Street, Danville 24541, VA, USA

**Keywords:** fullerene, metallofullerene, liposome, antioxidant, anti-inflammation, photosensitizer, drug delivery, molecular imaging, magnetic resonance imaging

## Abstract

Fullerene medicine is a new but rapidly growing research subject. Fullerene has a number of desired structural, physical and chemical properties to be adapted for biological use including antioxidants, anti-aging, anti-inflammation, photodynamic therapy, drug delivery, and magnetic resonance imaging contrast agents. Chemical functionalization of fullerenes has led to several interesting compounds with very promising preclinical efficacy, pharmacokinetic and safety data. However, there is no clinical evaluation or human use except in fullerene-based cosmetic products for human skincare. This article summarizes recent advances in liposome formulation of fullerenes for the use in therapeutics and molecular imaging.

## 1. Introduction

The discovery of a spherical crystal form of carbon, bound by single and double bonds that form a three-dimensional geodesic spheroidal crystal, named fullerenes or buckminsterfullerene, by Curl, Kroto, and Smalley in 1985 opened a new field of science—nanotechnology [1]. Fullerenes are hollow cage molecules with sp^2^ carbon atoms arranged in hexagons and pentagons. Since the discovery of C_60_, other fullerenes, including C_70_, C_72_, C_74_, C_76_, C_78_, and C_84_, have also been produced. The diameter of the fullerene cage is approximately 7–10 Å. Although their molecular weight is high, due to the high number of carbon atoms arranged in a spherical geometry, fullerenes are similar in size to most small organic molecules such as steroid hormones and peptide alpha helices—an important property from the drug design and development perspective. Because of their spherical shape and their one nanometer size, fullerenes are often denoted as buckyballs or carbon nanomaterials. On the contrary, fullerenes and the majority of their derivatives have a fixed molecular formula and elemental composition like other small molecules. The small volume of fullerene molecules is a very useful property for biomedical application, as the smaller size of nanoparticles is less likely to be taken up by the reticuloendothelial system compared to the larger conventional nanoparticles such as titanium dioxide and gold nanoparticles. Fullerenes have a uniquely delocalized π electron cloud across the cage surface with each carbon atom contributing one π electron, rendering fullerene with very high electron affinity. The absence of any reactive site on the cage surface except the carbon-carbon double bonds often makes the cage inert under physiological conditions. From the chemistry perspective, fullerenes are superconjugated, electron deficient poly-olefins. From the biology perspective, fullerenes are superpowerful antioxidants capable of scavenging and detoxifying reactive oxygen species (ROS) and reactive nitrogen species (RNS) [2,3,4,5,6].

**Figure 1:**
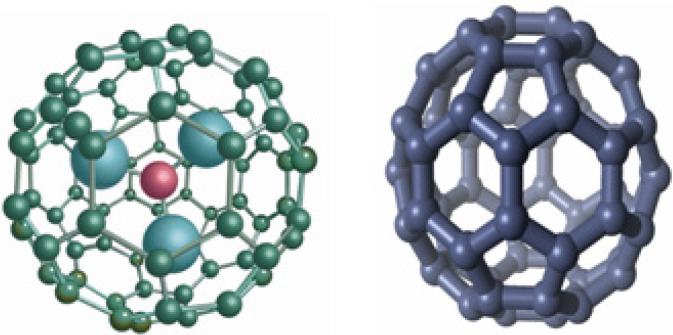
Structures of trimetallic nitride endohedral metallofullerene and empty cage fullerene C_70_.

Fullerenes have hollow interiors, where other atoms and ions can be entrapped. Those materials that encapsulate metal atoms are called endohedral metallofullerenes [7,8]. One important property of the endohedral metallofullerene is the highly stable encapsulation of potentially toxic metal ions or otherwise unstable metal clusters for therapeutic and diagnostic applications. There is no covalent or coordinate bond formation between the metal atoms with the carbon cage, but rather the metal atoms are physically trapped inside the cage. In some cases, depending on the size of the metal clusters, they can freely rotate within the cage. The metal inside the cage is not accessible for replacement such as transmetallation with endogenous ions, making it far more stable and inert than traditional metal chelates that are used in contrast-enhanced medical imaging. This property has been exploited recently in encapsulating gadolinium for use as MRI contrast agents, and radioactive isotopes for use in nuclear medicine including radiotherapy and nuclear imaging (PET and SPECT). Figure 1 shows the molecular structural of one representative metallofullerene—trimetallic nitride metallofullerenes M_3_N@C_80_, and empty cage fullerene—C_70_.

## 2. Biomedical Applications of Fullerenes

There are many excellent reviews on the biological and medical potentials of fullerenes [2,3,4,5,6,7]. A brief summary of biomedical applications of fullerenes is included in Table 1. Based on the role fullerenes play, their applications can be categorized into five groups: (1) Fullerenes act as potent antioxidants by scavenging free radicals including reactive oxygen species and reactive nitrogen species, and related applications include anti-aging agents that delay the normal aging process or extend the lifespan, neuroprotectants that treat neurodegenerative diseases such as Parkinson’s Disease (PD) and Amyotrophic Lateral Sclerosis (ALS), and radioprotectants that mitigate or prevent tissue and cell damages by ionizing radiation; (2) Fullerenes are nanoscale scaffolds for building novel molecular or particulate entities, where one or more functional groups are covalently attached to fullerene cage surfaces in a geometrically controlled manner. Applications include drug vector for delivering nucleic acids across biological membranes and receptor ligands for agonizing or antagonizing cellular and enzymatic processes; (3) Fullerenes are stable cage molecules that can encapsulate toxic or unstable atoms, ions or clusters inside the cage. Gadofullerenes are very promising MRI contrast agents where one or more gadolinium atoms are entrapped inside the fullerene cage to provide stronger gadolinium affinity and reduced long-term safety concerns associated with released Gd^3+^ from clinically approved Gd-chelate contrast agents; (4) Fullerenes are photosensitizers and can absorb photons in the ultraviolet and visible electromagnetic spectrum to produce photoexcited fullerene species in triplet state that subsequently transfer energy or electron to molecular oxygen leading to the generation of singlet oxygen or reduced oxygen species depending on the polarity of the medium. Fullerenes thus can be used in photodynamic therapy for treating cancer and killing microorganisms; (5) Fullerenes are excellent electron acceptors due to their high electron affinity, and can be used in building biosensors or enzyme-mediated biofuel cells.

**Table 1:** Summary of biomedical applications of fullerenes.

Fullerene role	Applications	Commercialization leaders	
Antioxidant; Free radical scavenger	Anti-aging,	The Bronx Project Inc.;	
	Neuroprotection	TDA Research Inc.;	
	Radioprotection	Vitamin C60 Bioresearch Corporation;	
	Cosmetics	Navya Biomedical Technologies LLC;	
Encapsulating toxic or unstable species	MRI contrast agent, Radiopharmaceuticals	Kepley Biosystems Incorporated;	
		Luna Innovations Incorporated (Luna acquired certain fullerene IPs originated from Tego Bioscience and C Sixty Inc.)	
Three dimensional nanoscale building block	Gene delivery		
	Transfection vector		
	Enzyme inhibition		
Photosensitizer	Photodynamic therapy,		
	Antimicrobials		
Electron acceptor	Biosensors		
	Biofuel cells		

Fullerenes and their derivatives have been proposed as free radical scavengers [9], and a number of investigations have studied fullerene derivatives as potential free radical antioxidant therapeutics, neuroprotectants, anti-inflammatories and others [5,10]. Fullerenes (both pristine and derivatized fullerenes) have a tendency toward aggregation in aqueous environments making them less desirable for pharmaceutical applications. For example, formulation techniques for preparing fullerene-based therapeutic candidates include host-guest complexation with cyclodextrins and calixerenes, surfactant solubilization with Tween-20 and polyvinylpyrrolidone (PVP), and so forth. These preparations have their respective limitations in terms of uniformity of formulation, loading capacity, aggregation, and partition coefficient. Derivatization of fullerenes by directly adding moieties to the carbon cage has been used as a strategy to produce useful drug candidates. Such fullerene compounds including polyhydroxylated C_60_ (fullerenol), polysulfonated C_60_, and carboxylated fullerenes have been shown to block free radical damages in several oxidative stress-related diseases including ischemia/reperfusion injury, inflammatory apoptosis, neurogenerative diseases, and asthma [11,12,13,14,15]. However, aggregation in aqueous media to form particles with a broad range of diameter distribution is a general problem for many of those compounds [16]. Polyderivatized fullerenes that have multiple moieties attached to the fullerene core are often a mixture of many compounds, poorly characterized and not suitable for pharmaceutical development. In addition, it has been shown that cytotoxicity of fullerenes is related to the degree of cage derivatization, water solubility, aggregation, and particle size [16]. An alternative approach in which fullerenes are encapsulated in bilayer vesicles such as liposomes has been proposed to overcome these limitations.

## 3. Structures and Compositions of Fullerene Liposome

**Figure 2:**
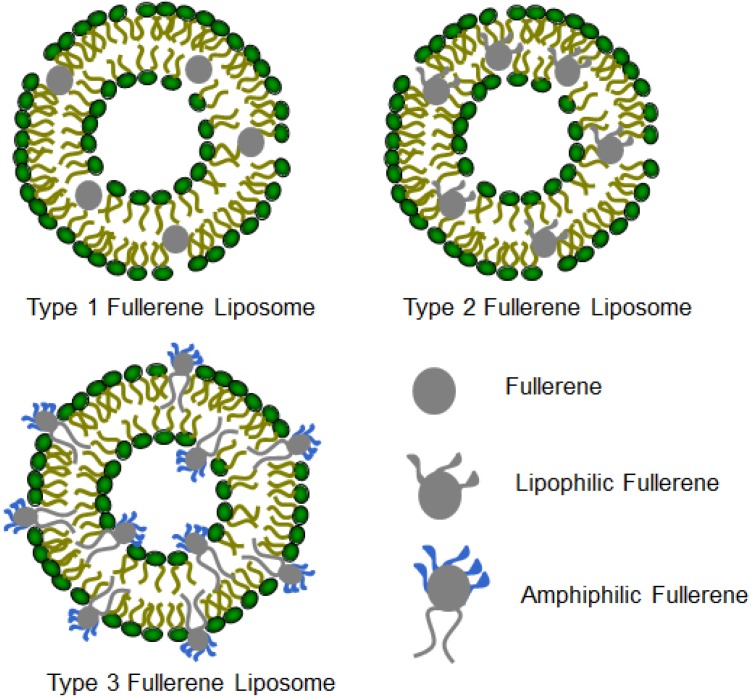
Three types of fullerene liposomes incorporating un-derivatized fullerenes, lipophilic fullerenes and amphiphilic fullerenes, respectively.

Drug delivery systems have become important tools for the delivery of a large number of drug molecules with enhanced therapeutic index or imaging sensitivity. Liposomes—nanosized unilamellar or multilamellar phospholipid vesicles—are versatile drug delivery systems for both hydrophilic and lipophilic molecules. Liposome formulated pharmaceuticals and cosmetics have been clinically approved for use in treating cancer, topical diseases, skin care and others. Therefore, formulating fullerenes in liposomes may create novel pharmaceutical and cosmetic products. The fullerene cage is entirely composed of carbon atoms, rendering them lipophilic and aromatic. Bensasson *et al*. described the preparation of vesicles by incorporating C_60_ into L-α-phosphatidyl choline purified from egg yolk (Egg-PC) [17]. The authors reported that 3% or less of C_60_ was incorporated in Egg-PC liposomes and the preparation was not uniformly reproducible. Incorporation of C_60_ into L-α-phosphatidyl ethanolamine (PE) was limited to 7% [18]. Initial efforts focused on the use of un-derivatized, spherical fullerenes that are structurally not compatible with natural phospholipids; therefore, the fullerene contents were low and their dimensional stabilities were problematic. Modification of the fullerene cage can change their physicochemical properties including lipophilicity and hydrophilicity. Covalent attachment of lipophilic moieties such as long alkyl molecules prepared highly lipophilic compounds that can be extremely soluble in non-polar organic solvents such as diethyl ether and chloroform [19,20,21,22]. The lipophilicity depends on the nature and the number of such moieties attached to the fullerene cage. On the other hand, two or more different moieties can also be added to the fullerene cage to form amphiphilic fullerenes, which can nicely co-assemble with lipids to form liposomes [23]. Three basic types of fullerene liposomes have been reported: type I fullerene liposome incorporates un-derivatized fullerene molecules; type II fullerene liposome incorporates lipophilically modified fullerenes; and type III fullerene liposome incorporates amphiphilic fullerene compounds (Figure 2). To synthesize fullerene amphiphiles, a variety of polar headgroups have been attached to the fullerene core with long alkyl chains as the lipophilic moieties. Common polar headgroups include carboxyls (e.g., malonic acid and glycolic acid), sugars (e.g., *myo*-inositol and phosphorylated inositol), glycerol, and positively charged ammonium salts (e.g., ethanolamine and dimethylethanolamine). These headgroups with different charges can further modulate the liposome interaction with biological molecules, cells and tissues to achieve desirable biodistribution properties.

## 4. Fullerene Interactions with Lipid Bilayers

Lipid bilayers can solubilize C_60_ and C_70_ fullerenes, and the fullerene loading within the bilayer depends on both the structure of lipids and the lipophilicity of fullerene compounds. Fullerenes without modification are barely soluble in non-polar, non-aromatic solvents such as diethyl ether and chloroform, which are widely used in the preparation of liposomes. Both experiments and simulations confirm that fullerenes partition to the membrane interior, although experimental information on the location of fullerene molecules is only qualitative [24,25]. On the other hand, the fullerene dispersion state is difficult to assess experimentally, and appears to depend on the details of the methodology used for the preparation of fullerene-loaded liposomes. Large aggregates observed in the presence of lipid membranes are unlikely to be found within the membrane, as they are orders of magnitude larger than the membrane thickness. There are some molecular dynamic simulations but a paucity of experimental evidence of the effects of C_60_ fullerene on lipid bilayers.

C_60_ suspension has previously been shown to provoke cell membrane destabilization *in vivo*. Phase contrast microscopy and computer aided image analysis results show that C_60_ causes shape transformations and rupture of unilamellar phospholipid vesicles, indicative of changes in their average mean curvature. Small-angle X-ray scattering reveals that C_60_ provokes disruptions of external membranes of multilamellar vesicles only after freeze and thaw cycles. The liposomes undergo breakage and annealing steps which increase the probability for fullerenes to insert into the MLVs. Experimental findings confirmed the potential of C_60_ to reconstruct lipids in biological membranes. Perturbation of lecithin bilayers by unmodified C_60_ fullerenes using experimental methods and computational simulations was also carried out [26]. Physicochemical and theoretical tools were used to understand fundamental problems of the interaction between lipid bilayers (Egg-PC liposomes) and unmodified C_60_ fullerenes. The morphology, size, and electrokinetic properties of plain and C_60_-loaded liposomes were investigated by means of atomic force microscopy, dynamic light scattering, and ζ-potential studies, respectively. The incorporation of C_60_ molecules into the liposomes increases their size; however, there was no effect on their electrokinetic properties. Fluorescence measurements showed that the presence of C_60_ in liposomes causes a pronounced effect on the Nile red emission spectrum due to alterations to the packing of the lipid membrane. The release of vesicle-encapsulated calcein was used as a measure of the integrity of the liposomes. Plain liposomes were found to be more stable compared with C_60_-loaded (PC) liposomes, suggesting that C_60_ ruptures the liposome membrane. Theoretical calculations show that low concentration of fullerene molecules present in the membrane had no effect on the membrane integrity; however, at high concentrations of fullerenes, significant enlargement of the surface area is observed. Thus, surface functionalized fullerenes with proper lipophilic, hydrophilic or amphiphilic groups are preferred to prepare fullerene derivatives for better fullerene liposome formulations.

A photocurrent generation system based on phospholipid-assembled fullerenes was also reported and it relies on the non-covalent formulation of a lipid bilayer on an electrode to achieve precise placement of fullerene. Photosensitizing dyes were added to the system and achieved higher conversion efficiency [27].

## 5. Fullerene Liposome Antioxidants

**Figure 3:**
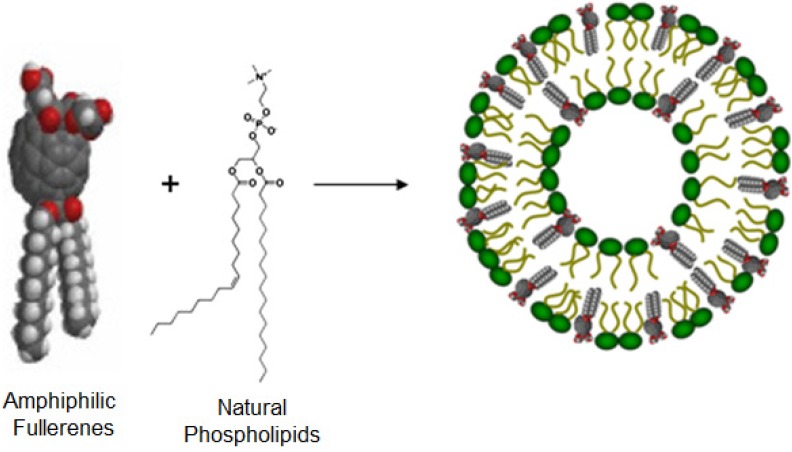
Vesicle formation of amphiphilic fullerene compound ALM with auxiliary phospholipids.

Given the low contents of pristine fullerenes in liposomal formulations, it is hypothesized that incorporating amphiphilic fullerenes in vesicles would greatly increase their ability to be intercalated within the lipid bilayers. Zhou *et al*. reported the design and synthesis of a new class of amphiphilic fullerenes, their liposome formulation and biological activities as free radical scavengers [24]. A key to obtaining a uniform vesicular preparation with high fullerene content and dimensional stability is to incorporate amphiphilic fullerene derivatives which mimic the structure of natural phospholipids. Figure 3 illustrates the design strategy in which the amphiphilic fullerenes and phospholipids co-assemble and form bilayer vesicles. This method leads to highly increased loading capacity of fullerenes. The amphiphilic fullerene form bilayer vesicles with lipid-to-fullerene molar ratio greater than 1:1 to produce uniform and dimensionally stable vesicles. They also reported that the oval structure of C_70_ molecule (as opposed to the spherical C_60_) provides a novel structural platform to prepare this new type of amphiphilic fullerenes. C_70_ has two reactive poles and a relatively inert equatorial region, and this allows for sequentially attaching lipophilic and hydrophilic groups at the two poles, respectively. The large un-derivatized zone around the C_70_ belt has very high radical reactivity due to the significant orbital overlap of its lowest unoccupied molecular orbital (LUMO) and highest occupied molecular orbital (HOMO) that is expected for sites of maximum radical reactivity [28,29,30]. The fullerene-enriched liposome provides a novel formulation approach that not only enhances the fullerene delivery efficiency, but also maintains their antioxidative properties. By mimicking the structure of naturally occurring lipid molecules, this design overcomes several limitations in fullerene liposome formulations including (1) low fullerene content when un-derivatized fullerenes are used and (2) damaged antioxidative bioactivity due to the additions of multiple groups to fullerenes. The strong association between amphiphilic fullerenes and auxiliary lipids allow them to form dimensionally stable liposomes with as high as 65% (by weight) fullerene. The antioxidant property of fullerenes is retained in the bipolarly functionalized C_70_ derivative, amphiphilic liposomal malonylfullerene (ALM), as well as in its liposomal formulations, as shown by both electron paramagnetic resonance (EPR) studies and *in vitro* reactive oxygen species inhibition experiments. The liposomally formulated ALM efficiently quenched hydroxyl radicals and superoxide radicals. In addition, the fullerene liposome inhibited radical-induced lipid peroxidation and maintained the integrity of the lipid bilayer structure. This new class of liposomally formulated, amphipathic fullerene compounds represents a novel drug delivery system for fullerenes and provides a promising pathway to treat oxidative stress-related diseases.

In order to increase the efficiency of delivery of fullerenes to target tissues, lipophilic fulleropyrrolidine derivatives Q-C_60_: [*N*-methyl-(2-quinolyl)fulleropyrrolidine] and I-C_60_: [*N*-methyl-(2-indolyl)fulleropyrrolidine] were also synthesized and encapsulated in multilamellar phospholipid liposomes, and the antioxidative capacity was studied using EPR spin-trapping and spin-labeling techniques [31]. Its capacity for removal of •OH (hydroxyl radical) and O_2_^•−^ (superoxide radical) and for the prevention of lipid peroxidation were compared with the performance of pristine C_60_, Q-C_60_ and I-C_60_ showed similar, or even better, antioxidative characteristics. Other fulleropyrrolidine derivatives were also reported for incorporation in liposomes [32].

## 6. Fullerene Liposomes Inhibit Inflammation

Inflammation is a natural biological response that occurs when vascular tissues are subjected to harmful stimuli. This process may be beneficial to the host during wound healing and infections but can be detrimental if left unchecked. Oxidative stress—the generation of reactive oxygen species—is thought to be one component of this response. Fullerenes can counteract reactive oxygen species due to their potent antioxidant capabilities. Thus, it was hypothesized that these molecules may inhibit inflammation. An *in vivo* model of phorbol 12-myristate 13-acetate (PMA)-induced inflammation was used and the effects fullerenes have on mitigating this response was investigated. PMA-induced inflammation and oedema were dramatically inhibited when liposomally formulated fullerenes were applied topically. Thus, fullerene derivatives may be a novel way to blunt certain inflammatory conditions and facilitate faster recovery of damaged tissue [33].

**Figure 4:**
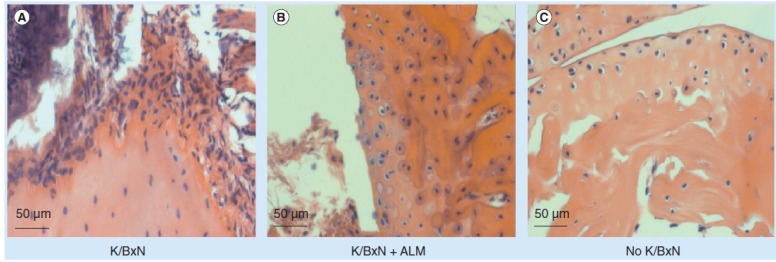
Fullerenes attenuate inflammatory arthritis of the K/BxN-induced disease pathology. (**A**) Serum-treated mice demonstrated typical synovial hyperplasia, pannus formation and inflammatory infiltrates; (**B**) By contrast, ALM-treated animals had less evidence of clinical joint inflammation compared with (**C**) non-diseased animals. ALM: Amphiphilic liposomal malonylfullerene.

Fullerene derivatives have also been shown to have anti-inflammatory capabilities through their ability to stabilize Mast Cells (MC) preventing inflammatory mediator release [34]. Given that MC function as a cellular link between autoantibodies, soluble mediators, and other effector populations in inflammatory arthritis, it is hypothesized that fullerene derivatives could inhibit and/or reverse this inflammatory disease. A panel of fullerene derivatives was tested for their ability to stabilize MC mediator release in response to FcγR-dependent mediator release, inhibit osteoclast formation, and prevent cytokine production from human synovial fibroblasts [35]. It is shown that certain fullerene derivatives blocked FcγR- and TNF-α-induced mediator release from skin derived MC and TNF-α-induced mediator release from synovial fibroblasts (RA patients), as well as the formation of human osteoclasts. The MC-inhibition by fullerene derivatives was mediated through the inhibition of mitochondrial membrane potential and FcγR-mediated increases in cellular reactive oxygen species (ROS) and NF-κB activation. Based on the *in vitro* data, two fullerene derivatives (ALM and TGA) were selected for *in vivo* studies. Fullerenes inhibited the edema, inflammation, cartilage/bone erosion, and TNF-α levels associated with arthritis in K/BxN serum transfer arthritis but not in collagen-induced arthritis (Figure 4). The anti-arthritis fullerene derivative ALM labeled with infrared dyes was able to localize to the affected joint areas in diseased animals but not in healthy control animals. An IR-800 dye was conjugated to the hydrophilic moiety of ALM and formulated in liposome. The results demonstrated that the liposome formulation of amphiphilic fullerene ALM is capable of preferentially accumulating in the inflamed synovial joints. At day seven post serum (or vehicle) injection, during the peak symptom scores, dye-labeled ALM is clearly visible six hours post injection in the joints of mice with inflammatory arthritis. In contrast, control mice without inflammatory arthritis receiving the same dose of fullerene-dye conjugates did not demonstrate fullerene-dye accumulation in the joints. These data confirm that ALM is capable of accumulating within the joints of mice with “active” inflammatory arthritis where they are poised to inhibit the inflammatory cascade [35].

## 7. Fullerene Liposome for Photodynamic Therapy

Photodynamic therapy (PDT) uses non-toxic photosensitizers that absorb photons to produce a long-lived triplet state that kills cells in the presence of molecular oxygen. Fullerene liposomes have been reported to act as a photosensitizer in biological systems. Fullerene liposomes were prepared by transferring fullerenes from their water-soluble host-guest complexes (C_60_·γ-CD and C_70_·γ-CD) to lipid membranes for photodynamic therapy [36,37]. The fullerene-exchange reaction from a cyclodextrin cavity to liposomes occurred completely at temperatures above the phase transition temperature (T_m_) of the liposomes; however, lowering the temperature to below the T_m_ led to C_60_ or C_70_ aggregation outside the liposomes. The exchange reaction was also found to occur more efficiently by the addition of small amounts of unsaturated lipids bearing a π-moiety, which acts as a gate when hydrophobic fullerene migrates into the hydrophilic liposome surface. Ikeda *et al*. reported various types of lipid membrane-incorporated C_60_ with high C_60_ concentrations prepared using the exchange method, where fullerene C_60_ was first solubilized by complexing with gamma-cyclodextrin following guest fullerene molecule exchange from cyclodextrins to lipid bilayers, and the photocleavage activity of the resulting cationic lipid membrane-incorporating C_60_ was appreciably higher than that of the C_60_-cyclodextrin complex [37]. Singlet oxygen was generated effectively by photoexcited C_60_ in nonpolar solvents such as benzene and benzonitrile, and it was found that superoxide radical anions and hydroxyl radicals were produced instead of singlet oxygen in polar solvents such as water, especially in the presence of a physiological concentration of reductants including NADH. Results of DNA cleavage assays in the presence of various scavengers of specific active oxygen species indicate that the active oxygen species primarily responsible for photoinduced DNA cleavage by C_60_ under physiological conditions are reduced species superoxide radical anion and hydroxyl radicals [38,39]. Liposomes incorporating fullerene C_70_ by the same exchange method was also reported for efficient DNA photocleavage. Fullerene PDT can also be used to save the life of mice with wounds infected with pathogenic Gram-negative bacteria and to treat mouse models of various cancers. Fullerene-liposome was also reported to reduce mean pulmonary virus yields, decrease the lung index and significantly prolong mean time to death (MTD) and decrease mortality of H1N1 virus-infected mice, suggesting that fullerene-liposome has the anti-influenza activity *in vivo* at much lower concentrations as compared to the Rimantadine [40]. *In vivo* photodynamic therapy with fullerene liposomes or other water soluble fullerene compounds may represent a new application in fullerene nanomedicine.

## 8. Metallofullerene Liposome for Contrast-Enhanced Molecular MR Imaging

**Figure 5:**
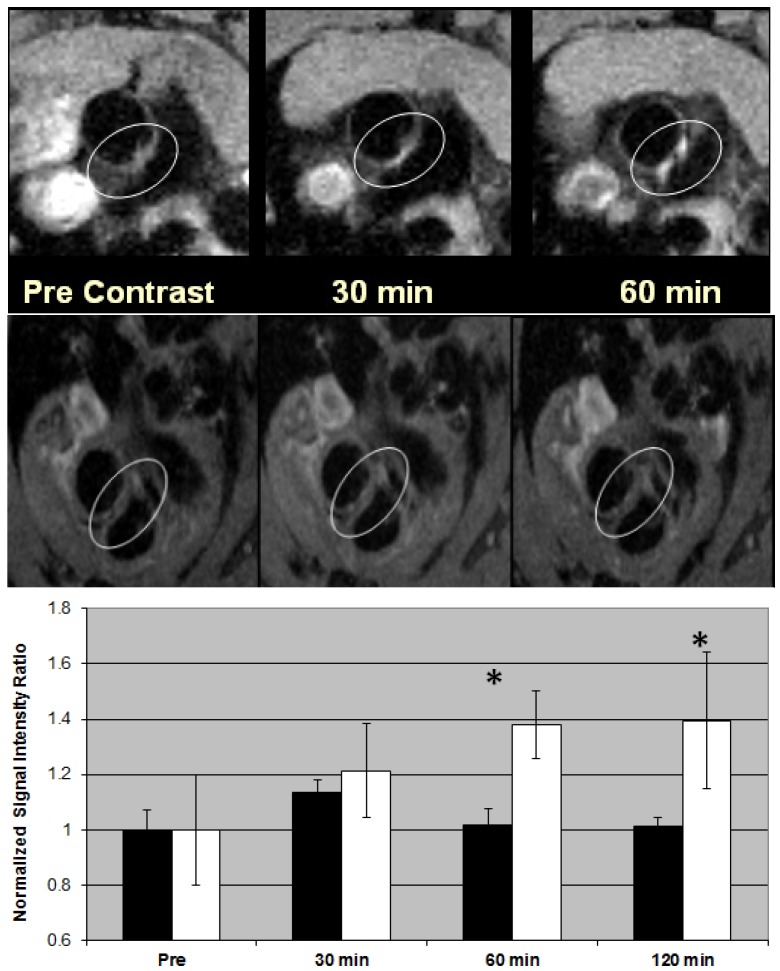
Figure: Plaque imaging of aorta arch of 23 weeks old female ApoE^−/−^ mice with 7T imager. **Top**: ATCA (100µL, 4.0 mg/kg or 0.9 mg Gd/kg); **Bottom**: Non-targeted control did not enhance any contrast. Graph: Quantification of signal enhancement. The signal to noise ratio (SNR) was calculated using a reference ROI next to the plaque lesion, then SNR values were normalized to the pre-injection SNR value. The same regions were traced throughout all imaging points. ATCA: white bars, and non-targeted control: black bars. The SNR values are shown as mean for the entire aorta of five mice. * indicates significance (*p* < 0.05).

Dellinger *et al*. reported the synthesis and imaging study of a liposome formulated gadofullerene MRI contrast agent for targeted imaging of macrophage receptors for the diagnosis of unstable atherosclerotic plaque [41]. Atherosclerosis-targeting contrast agent (ATCA) incorporates a recently discovered metallofullerene MRI T_1_ contrast agent (Hydrochalarone^®^) and the specially oxidized lipid 1-palmitoyl-2-arachidonoyl-*sn*-glycero-3-phosphocholine (oxPAPC) targeting inflammatory macrophage CD36 receptors in a self-assembled liposome construct [42]. Amphiphilic Hydrochalarones^®^ were co-assembled with natural phospholipids and oxPAPC (type III fullerene liposome). OxPAPC is a biologically active oxidized phospholipid first isolated from minimally oxidized low density lipoproteins, known to be internalized by macrophages via the CD36 scavenger receptor. Inflammatory macrophages participate in all phases of atherogenesis including lesion initiation, progression, and complications, and have been validated as viable targets for molecular imaging [43]. ATCA exhibited time-dependent selective accumulation and significant MR detectable contrast enhancement in atherosclerotic plaque lesions of ApoE^−/−^ mice at ~5% of the gadolinium dose of clinically used T_1_ agents [41]. As seen in Figure 5, ApoE^−/−^ mice (23 weeks, 5 mice per group) injected *i.v.* with ATCA (100 μg/100 μL or 0.9 mg Gd/kg; clinical dose for Omniscan or Magnevist is 10–20 mg Gd/kg) had a noticeable enhanced image of the plaque lesions attached in the aorta arch that could not be seen prior to injection or when imaged with non-targeting control agent (without oxPAPC in the formulation). The ratio of signal intensity of the whole region lining the aorta wall to that of the (non-affected) myocardium at 60–120 min post injection was compared to the pre-contrast ratio showing ~67% signal enhancement. Whole-body imaging did not show any measurable uptake in major organs. When ATCA was injected to atherosclerosis-free wild-type C57 mice, the aorta imaged at up to 4 h did not demonstrate signal enhancement. Histologic evaluation revealed co-localization of the ATCA agents with macrophage foam cell-rich regions of the plaque, whereas little or no uptake was observed in non-inflammatory cells. These *in vivo* results were consistent with cell culture observations. Atherosclerotic plaques with significant imaging enhancement were found to have CD36 receptor-positive cells as shown by immunostaining. The hypothesis that a self-assembled liposome construct is capable of selectively delivering the imaging agents to macrophage present in plaque lesions for binding and internalization was also verified by Western blotting analysis of CD36 receptor-specific signaling pathways, by flow cytometry analysis and by focused ion beam/scanning electron microscopy that visualize the Gd-containing metallofullerene nanoparticles inside macrophage [41].

These results demonstrated the ability of ATCA to image a specific molecular or cellular process of atherogenesis using a liposome formulation. ATCA is particularly suited to image plaque due to its oxidized LDL mimetic characteristics. The specificity of the *in vivo* targeting has been shown to be very high. ATCA liposome particles penetrate through the highly permeable endothelium overlying atherosclerotic plaques to target macrophages. In other organs and tissues such as those present in the reticuloendothelial system, ATCA particles need to extravasculate through the tight endothelium in order to become accessible to macrophages. During this process the liposome construct often dissociates upon internalization and releases the amphiphilic contrast agents and the oxidized phospholipids as two separate entities. Liberated oxPAPC is thus no longer capable of directing the imaging agents to macrophages. These characteristics enhance the specificity of labeling to atherosclerotic plaque macrophages. Due to the extremely low dose of ATCA used for imaging it is less likely to be detected by MRI unless ATCA is accumulated in specific tissues. MR imaging of murine plaques was also reported by using gadolinium chelates-loaded micelles (particle relaxivity of ~2 × 10^5^ mM^−1^ s^−1^) targeting macrophage receptors [44]. Experimental data and theoretical predictions made by Tweedle and others have suggested that to achieve a 50% increase in image contrast, the minimum concentration is 5 × 10^−7^, 5 × 10^−8^, and 1.6 × 10^−11^ moles per gram of tissue for extracellular Gd-chelate agents (e.g., Gadoteridol), albumin-binding blood pool agents (e.g., Gadofosveset), and superparamagnetic iron oxide particles (e.g., SPIO), respectively [45]. ATCA has unprecedented high relaxivity. Hydrochalarone^®^ has a molecular relaxivity of 183 mM^−1^ s^−1^ in plasma at 1.5 T, over 40-fold better than Magnevist. Since one ATCA liposome nanoparticle contains roughly 30,000 Hydrochalarone^®^ molecules, ATCA has a particle relaxivity of about 5.49 × 10^6^ mM^−1^ s^−1^. Thus, its concentration in plaque needs to be at least 3 × 10^−12^ moles per gram issue in order to achieve 50% enhancement. Since the CD36 receptor is expressed on macrophages in the range of 10^−10^–10^−^^12^ mol/g. It is feasible to label macrophages with sufficient numbers of contrast agents to produce significant contrast enhancement.

## 9. Fullerene Liposome for Chemotherapy and Cancer Theranostics

Fullerenes have also been developed as slow-release systems for liposome aerosol delivery of lipophilic chemotherapeutics. Paclitaxel, one of the most active anticancer drugs in clinical use, has rapid clearance from the lungs (within 40 min after cessation of aerosol delivery) resulting in reduced therapeutic efficacy. A C_60_-Paclitaxel conjugate was synthesized and formed a stable liposome formulation with dilaurylphosphatidylcholine (DLPC) [46]. The conjugate was designed to have no activity by modifying 2'-hydroxyl group of Paclitaxel and to release the active drug via enzymatic hydrolysis of a 2'-ester bond in plasma with the hydrolysis half-life of about 80 min. The formulation demonstrated cytotoxic activity comparable to that of Paclitaxel in human epithelial lung carcinoma A549 cells. With both clinically-relevant kinetics of hydrolysis and significant cytotoxicity in tissue culture, the fullerene-Paclitaxel conjugate holds promise for enhanced therapeutic efficacy of Paclitaxel.

Multifunctional nanoparticles that incorporate receptor ligand, imaging probe and therapeutic payload are called theranostics and could be the future of medicine, and fullerenes prove to be an attractive platform for these applications. Metallofullerene MRI contrast agents have been incorporated in liposome formulations with cancer chemotherapeutic agents as brain cancer theranostics. Glioblastoma multiforme is an aggressive high-grade brain tumor with a poor prognosis. Ideally, a multifunctional nanoscale compound is needed that can simultaneously diagnose tumor progression and can also specifically target and kill the tumor. IL-13 receptors are selectively expressed on astrocytoma cells in gliomas. MRI using gadolinium-based contrast agents has been used for diagnosing glioblastoma. Amphiphilic Hydrochalarones were used as a platform for developing novel glioblastoma-targeting theranostics in which the diagnostic agent is enclosed within glioblastoma-targeting IL-13 liposomes that can deliver a therapeutic payload of doxorubicin that was encapsulated within the lipid bilayer. After verifying *in vitro* binding of the glioblastoma-targeting theranostics to human glioblastoma cells, the nanoparticles were tested *in vivo*. The glioblastoma theranostic can target and shrink human brain tumors that have been transplanted in mice [35].

## 10. Fullerene Liposomes for Cosmetics

Reactive radical species are believed one of the causes of various skin problems. These radicals that are produced upon exposure to ultraviolet radiation can lead to the production of melanin that causes flecks and freckles, wrinkle formation, and skin irritation. Antioxidants such as Vitamin C and Vitamin E are known to be effective to remove and detoxify these damaging radicals. Thus, it is proposed that these skin problems may be mitigated by applying fullerenes with superpowerful antioxidative potency at least two orders of magnitude greater than vitamins. Fullerene incorporated in liposome exerts persistent hydroxyl radical scavenging activity and cytoprotection in UVA/B-irradiated keratinocytes [47]. The fullerene liposome is composed of hydrogenated lecithin of 89.7%, glycine soja sterol of 10%, and C_60_ of 0.3%. Hydroxyl radicals generated from UVA- or UVB-irradiated H_2_O_2_, were scavenged by the fullerene liposome but not by C_60_-lacking liposome controls, showing that the active principle is fullerene. Cell viability of human skin keratinocytes HaCaT decreased to 41.1% upon UVA-irradiation at 10 J/cm^2^, but retained 60.6% with 0.025% fullerene liposome (C_60_: 0.84 μM) together with prevention of cell-morphological degeneration. The scavenging activity for Fenton reaction-generated •OH, detected by DMPO/ESR, was 96.2% or 72.2% (% of control) at one minute and decreased time-dependently to 24.8% or 28.3% at 12 min with 16.7 μM L-ascorbic acid or Trolox, respectively, whereas 0.5% fullerene liposome (C_60_: 16.7 μM) diminished •OH by 90.9% at 1 min and 91.5% at 12 min, demonstrating the superiority of fullerene liposome to L-ascorbic acid or Trolox. Defensive effects of fullerene/liposome complex was also observed against UVA-induced intracellular reactive oxygen species generation and cell death in human skin keratinocytes HaCaT, associated with intracellular uptake and extracellular excretion of fullerene. The same fullerene liposome was also shown effective against UVA-induced damages in skin structure, nucleus and collagen type I/IV fibrils, and the permeability into human skin tissue [48,49].

## 11. Fullerene Buckysome

Amphiphilic C_60_ derivative AF-1 in which multiple aliphatic hydrocarbons were attached at various sites on the fullerene cage has been reported to self-assemble to form stable spherical nanometer sized vesicles [50,51]. The amphiphilic fullerene monomer (AF-1) consists of a “buckyball” fullerene cage to which a Newkome-like hydrophilic dendrimer unit and five lipophilic C12 chains positioned octahedrally to the dendrimer unit are attached. By inducing AF-1 self-assembly at an elevated temperature of 70 degrees, dense spherical buckysomes predominantly with diameters of 100–200 nm were formed, as observed by cryogenic electron microscopy, transmission electron microscopy, and dynamic light scattering. The amphiphilic nature of AF-1 results in the formation of many hydrophobic regions within the buckysomes, making them ideal for embedding hydrophobic molecules to be tested in a drug delivery scheme [52,53,54]. Thus, buckysomes are fullerene-based nanocarriers for hydrophobic molecule delivery. The unique functionalization of AF-1 permits the creation of buckysome structures that might offer advantages over traditional phospholipid liposomes as nanovectors. Liposomes are mostly suitable for carrying a hydrophilic payload in their hydrophilic compartment and less suitable for hydrophobic materials.

Cellular internalization of buckysomes embedded with the hydrophobic fluorescent dye 1,1'-dioctadecyl-3,3,3',3'-tetramethylindocarbocyanine perchlorate was observed. Paclitaxel, a highly hydrophobic anticancer drug was successfully encapsulated in buckysome. The *in vitro* therapeutic efficacy of the paclitaxel-embedded buckysomes toward suppression of MCF-7 breast cancer cell growth was compared to that of Abraxane, a commercially available, nanoparticle-albumin-bound formulation of paclitaxel. Notably, the paclitaxel-embedded buckysomes demonstrated a similar efficacy to that observed with Abraxane in cell viability studies; these results were confirmed microscopically. Moreover, negative control studies of MCF-7 viability using empty buckysomes demonstrated that the buckysomes were not cytotoxic. The results suggest that buckysomes prepared from self-assembly of AF-1 at 70 °C are promising nanomaterials for the delivery of hydrophobic molecules. Paclitaxel-embedded buckysomes have the potential for delivering the hydrophobic drug directly to tumor sites [52,53,54].

## 12. Summary and Perspectives

Fullerenes, often dubbed carbon nanomaterials, are indeed small molecules, similar in molecular size and volume to steroids, making them ideal molecular platforms to serve as ligands to enzymes and receptors. Other fundamental physical and chemical properties of fullerenes also govern how they may be adapted for biological use. Fullerene itself has no solubility in water; surface functionalization has led to a number of well characterized and studied compounds. However, fullerene derivatives often form aggregates in aqueous media, which could be problematic. The molecular character of fullerene compounds and their nanoparticulate behavior in aqueous media certainly will complicate the preclinical and clinical evaluations due to the dramatic difference in regulations for small molecule drugs and nanoparticle pharmaceuticals. Liposome formulation provides an alternative route to prepare fullerenes for pharmaceutical applications with enhanced distribution, absorption and delivery efficiency. A significant amount of scientific knowledge on fullerene medicine has been generated, yet there has been lack of progress in clinical studies, in part due to the concerns regarding long-term safety and toxicity in the pharmaceutical and investment community. With fullerene-based cosmetic products being clinically tested and used in human skincare for many years, it appears to suggest that at least topical application of fullerenes are safe. More information on the long-term toxicity of fullerenes that is well-purified and characterized is expected to eventually lead to broader clinical applications.
